# Dicer Functions in Aquatic Species

**DOI:** 10.4061/2011/782187

**Published:** 2011-06-09

**Authors:** Yasuko Kitagishi, Naoko Okumura, Hitomi Yoshida, Chika Tateishi, Yuri Nishimura, Satoru Matsuda

**Affiliations:** Department of Environmental Health Science, Nara Women's University, Kita-Uoya Nishimachi, Nara 630-8506, Japan

## Abstract

Dicer is an RNase III enzyme with two catalytic subunits, which catalyzes the cleavage of double-stranded RNA to small interfering RNAs and micro-RNAs, which are mainly involved in invasive nucleic acid defense and endogenous genes regulation. Dicer is abundantly expressed in embryos, indicating the importance of the protein in early embryonic development. In addition, Dicer is thought to be involved in defense mechanism against foreign nucleic acids such as viruses. This paper will mainly focus on the recent progress of Dicer-related research and discuss potential RNA interference pathways in aquatic species.

## 1. Introduction

**Figure 1:**
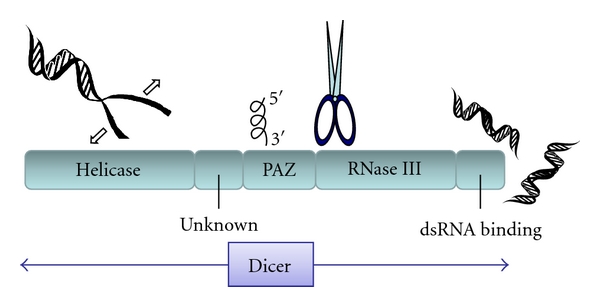
Schematic representation of the predicted consensual domain structure for the Dicer protein. Helicase: N-terminal and C-terminal helicase domains. PAZ: Pinwheel-Argonaute-Zwille domain. RNase III: bidentate ribonuclease III domains.

In eukaryotes, small RNA-mediated RNA silencing called RNA interference (RNAi) is able to suppress gene expression. Dicer is the key enzyme of the RNAi pathway to cleave double-stranded RNA (dsRNA) into small RNAs categorized as small interfering RNAs (siRNAs) or micro-RNAs (miRNAs), which are mainly involved in invasive nucleic acid defense and endogenous genes regulation, respectively [1–3]. Then, Dicer is reported to participate in both the antiviral immune response and developmental regulation. For example, Drosophila harboring the Dicer mutant exhibited enhanced disease susceptibility to cricket paralysis virus [4, 5]. In addition, Caenorhabditis elegans harboring the Dicer mutant had developmental phenotype defects [6–8]. The meRNAi is a conserved eukaryotic gene silencchanism that works at both the transcriptional and the posttranscriptional levels [9]. We fortuitously cloned and sequenced the human Dicer, (initially designated as HERNA) for the first time [10]. Dicer belongs to the RNase III family with ATP dependent RNA helicase, PAZ (Piwi/Argonaute/Zwille), dsRNA binding, and RNase III domains (Figure 1), which is responsible for cleaving long dsRNAs into siRNAs or miRNAs when associated with other proteins likeR2D2in Drosophila or the transactivating response RNA-binding protein in Homo sapiens to recruiting Argonaute proteins. The PAZ domain binds the single stranded 3′ end of small RNA [11], and it might function in protein-protein interaction. Small RNAs includes PIWI-associated RNAs (piRNAs), short single-stranded RNAs arising from a Dicer-independent pathway, which are found in germ cells and associate with the PIWI subfamily of Argonaute proteins [12, 13]. Many zebrafish piRNAs are derived from repetitive sequences. Mutations in the Piwi homologue protein result either in loss of germ cells or in defects in meiosis and chromosome segregation in eggs. However, the Dicer knockout mouse eggs raise a question about overlapping functions of vertebrate miRNA, RNAi, and piRNA pathways.

Most vertebrates, Urochordata, and worms are reported to have only one Dicer-1 protein, which generates both miRNAs and siRNAs. While insects, fungi, and plants have more than one Dicer or Dicer-like proteins [14], Dicer enzymes in Drosophila melanogaster are classified into Dicer-1 and Dicer-2 in terms of their specialized functional activities. Dicer-1 can process loop pre-miRNA to mature miRNA, while Dicer-2 can process dsRNA precursors into siRNAs molecules [15]. Recent studies have demonstrated that Dicer can function in an RNAi pathway independent manner. The Dicer-2 of Drosophila melanogaster participated in antiviral responses by mediating induction of antiviral gene Vago [16]. The Dicer-1 of Caenorhabditis elegans participates in fragmenting chromosomal DNA during apoptosis, and undergoes a protease-mediated conversion from a ribonuclease to a deoxyribonuclease in addition to the processing of small RNAs [17].

Since the initial discovery in 1998 by Fire et al. [18], RNAi has taken the biological community by storm. Despite many advances, however, RNAi is still under development. A better understanding of the mechanism for Dicer pathway is a future goal for many scientists. This paper will mainly focus on the recent evidences of Dicer functions in aquatic species. We will also highlight the effects of RNAi in experimental models of the aquatic species.

## 2. Expression and Developmental Function of Dicer in Aquatic Species

**Figure 2:**
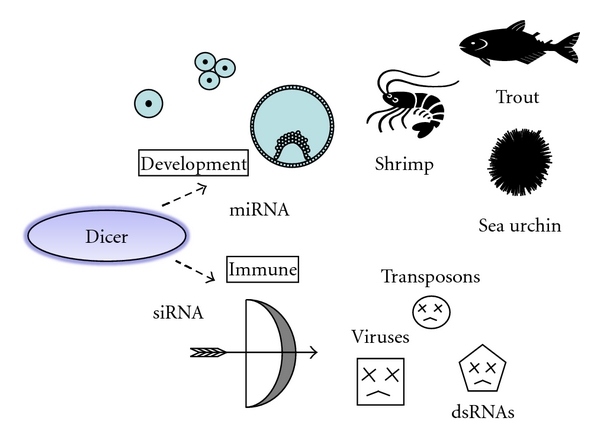
Small RNAs-dependent functions of Dicer. Schematic illustrations of the tentative model for developmental and immuno logical functions of Dicer in aquatic species are shown.

Only one homolog of Dicer was identified from the sea urchin, suggesting that the sea urchin Dicer may mediate both miRNA and siRNA-silencing pathways, similar to humans. The Dicer mRNA accumulates asymmetrically in one periphery of the oocyte in punctate cytoplasmic structures [19, 20]. The asymmetric localization of Dicer mRNA is maintained throughout the development of blastula and gastrula stages. The Dicer transcript accumulation is enriched selectively in the presumptive oral ectoderm and endodermal epithelium. The transcript then decreases to undetectable levels in the larval pluteus stage. Knockdown of Dicer in sea urchin embryos results in anomalous morphogenesis, such as impairment of gastrulation and skeletogenesis at the mesenchyme blastula stage and later stages, suggesting that miRNA could be involved in the early development of sea urchin [20]. Similarly, the Dicer transcripts in rainbow trout are detectable throughout the embryonic stages. Peak expression of Dicer at the time of maternal mRNA degradation and initiation of embryonic genome activation could indicate its involvement in miRNA processing during the periods in the rainbow trout [21] (Figure 2).

During the developmental stages from fertilized egg to postlarva, shrimp (*Litopenaeus vannamei*) Dicer-1 is constitutively expressed at all developmental stages [22]. The highest expression is observed in fertilized eggs and followed a decrease from fertilized egg to nauplius stage. Then, the higher levels of expression are detected at the late nauplius and postlarva stages. The shrimp Dicer-1 expression regularly increases at the upper phase of nauplius, zoea, and mysis stages than their prophase. The different expression in the larval stages might provide clues for understanding the early innate immunity in the process of shrimp larval development. The expression level of shrimp Dicer-1 mRNA varies significantly among different shrimp tissues. The expression in hemocyte is significantly higher than that in gill, muscle, brain, intestine, and pancreas. On the other hand, expression levels of shrimp Dicer-2 are about the same in most tissues, except in muscle, which has a lower expression level [23].

## 3. Immunological Function of Dicer in Aquatic Species

RNAi is a mechanism of posttranscriptional gene silencing that functions as a natural defensive response to viral infection in a variety of species (Figure 2). Knockdown of Dicer-1 in tiger shrimp (*Penaeus mondon*) resulted in increasing mortalities and higher viral loads, suggesting that the RNAi mechanism is active and has a powerful immunological function in shrimp [24]. Higher levels of Dicer-1 expression in lymphoid organs are consistent with a role in the natural defense response of shrimp. However, there is no correlation between levels of Dicer-1 expression and the viral genetic loads in shrimp lymphoid organ tissue during naturally acquired or persistent viral infections [25]. The Dicer-1 expression might be induced at an early stage of infection and recovers to normal levels later. 

The white shrimp (*Litopenaeus vannamei*) Dicer-2 involves in the nonspecific antiviral immunity, and in some degree supporting the suggested relationship between nonspecific activation of antiviral immunity and induction of RNAi [23]. The Dicer-2 might be contributed to nonspecific activation of antiviral immunity in shrimp by enhancing RNAi potency and efficacy [26]. The shrimp Dicer-2 might regulate the single von Willebrand factor type C domain protein genes in various ways [23]. Further research is required to identify more components of the shrimp RNAi pathway and investigate the exact mechanisms of genes regulation.

Targeted gene silencing and more potent, virus-specific immunity against challenge with white spot syndrome virus (WSSV) or Taura syndrome virus (TSV) have been obtained in Pacific white shrimp (*Penaeus vannamei*) by injection of long dsRNAs corresponding to sequences encoding viral structural proteins [27]. Similar virus-specific effects have been observed in *Penaeus monodon* shrimp using long dsRNAs targeting the yellow head virus (YHV) protease gene. However, siRNAs have failed to induce sequence-specific antiviral protection against WSSV or TSV. Similarly, inhibition of YHV replication in primary cell cultures appears to be less efficient when short dsRNAs are employed. Although RNAi has been assumed to be the mechanism of sequence-specific gene silencing and virus inhibition in shrimp, the interplay mechanism seems to be complex.

In many unicellular organisms, they seem to have retained only a basic set of components of the RNA-silencing machinery. *Chlamydomonas reinhardtii*, a unicellular eukaryote, has undergone extensive duplication of Dicer and Argonaute polypeptides after the divergence of the green algae [28]. Chlamydomonas encodes three Dicers and one of them is uniquely involved in the posttranscriptional silencing of retrotransposons. Then, multiple and redundant epigenetic processes are involved in preventing transposon mobilization in this green algae [28, 29].

## 4. Against Dicer Function

RNA silencing is now a well-known mechanism by which plants and invertebrates fight off viral infection; however, many plant and animal viruses possess proteins that suppress host RNA silencing mediated by siRNA or miRNA pathways. Striped jack nervous necrosis virus (SJNNV), which infects fish, has a bipartite genome of positive-strand RNAs. The SJNNV protein B2 has a potent RNA-silencing suppression activity [30]. Betanoda viruses are small RNA viruses that infect teleost fish and pose large threat to marine aqua-culture production. These viruses also possess the small protein B2, which binds to and protects dsRNA [31]. This prevents cleavage of virus-derived dsRNAs by Dicer and subsequent production of siRNA, which would otherwise induce an RNA-silencing response against the virus [32, 33]. The B2 is able to induce apoptosis in fish cells without dsRNA binding. The same tendency has been demonstrated using an RNA-silencing system in human HeLa cells [34].

The suppression mechanisms of RNA silencing by the viral suppressors have been widely investigated. Cucumber mosaic virus- (CMV-) encoded 2b protein (CMV2b) blocks miRNA pathways in green alga *Chlamydomonas reinhardtii* [35]. The CMV2b is able to bind small RNAs, suggesting that this may be a mechanism by which CMV2b interferes with RNA silencing. The CMV2b may suppress both siRNA and miRNA pathways in *Chlamydomonas reinhardtii*. The CMV2b containing an arginine-rich region has been reported to possess the ability to bind small RNAs. So, it is possible that CMV2b suppresses RNA-silencing pathways by directly interacting with small RNAs.

## 5. Perspectives

The miRNA pathway has been shown to be crucial in embryonic development and in embryonic stem (ES) cells, as shown by Dicer knockout analysis in mammals. Specific patterns of miRNAs have been reported to be expressed only in ES cells and in early phases of embryonic development. The miRNAs have emerged as key regulators of stem cells, collaborating in the maintenance of pluripotency, control of self-renewal, and differentiation of stem cells [36]. It has been demonstrated that Dicer is essential for the regulation of chondrocyte proliferation and differentiation during normal skeletal development in mice. Dicer deficiency in chondrocytes results in a reduction in the number of proliferating chondrocytes through decreased proliferation and accelerated differentiation into hypertrophic chondrocytes [37, 38]. Similarly, Dicer in mammals is involved in a lot of tissue-developmental stages such as limb [39], lung [40], retina [41], vessel [42], and female reproductive system [43], and so forth.

The development and use of RNAi techniques in basal metazoan model systems such as cnidarians will help to determine the evolutionary lineage and complexity of homologous pathways such as apoptosis in higher metazoans [44, 45]. The use of *acasp* RNAi will also enable us to answer questions about the role of cnidarian apoptosis in the onset and breakdown of symbiosis. This technique has been used in large-scale experiments in which manipulation of apoptosis resulted in a marked effect on symbiosis stability. 

Worldwide viral diseases by WSSV cause large-scale mortalities and substantial economic losses to shrimp aquaculture. The control of viral diseases in shrimp remains a challenge for the shrimp aquacultural industry. As shrimps lack adaptive immunity and the typical interferon response, RNAi is thought to be an ancient and important immune mechanism against virus replication. As mentioned before, the original function of RNAi is thought to be involved in defense mechanism against foreign nucleic acids, such as viruses, and in endogenous transcriptional regulation [2]. Presently, RNAi is becoming attractive to develop as an important potential tool in viral disease prevention in shrimp [46]. RNAi technology shows considerable promise as a therapeutic approach and efficient strategy for virus control in insects [47, 48]. Successful use of an RNAi technique for gene knockdown in the symbiotic sea anemone (*Aiptasia pallida*) has also been reported [49]. 

The use of RNAi technology in shrimp in vivo has indicated that the RNAi-effective time is limited due to the very short half-life of synthetic RNA duplexes. It is likely that siRNAs will not be present for complete elimination of virus replication at an infected tissue. In order to use RNAi technology on an effective manner to protect shrimp against viral diseases, it would be essential that future studies focus on increasing the stability of siRNA, and the relationship between the expression of antiviral immune genes in the immune response and larval development could shed light on the further practical application. However, this seems to be expensive approach yet. Instead, making transgenic shrimp which express an anti-viral shRNA might be a feasible way to engineer shrimp to be resistant to the bad viruses. More research on the characterization of RNAi-related genes in immune response and larval development may be helpful for better understanding the antiviral mechanism and designing efficient strategies of viral disease control. And the further progress in understanding the mechanism of RNAi will definitely revolutionize therapeutic approaches for counteracting diseases in aquatic species.

##  Conflict of Interests 

 The authors declare that they have no conflict of interests.
